# Factors that impact on women's decision‐making around prenatal genomic tests: An international discrete choice survey

**DOI:** 10.1002/pd.6159

**Published:** 2022-04-30

**Authors:** James Buchanan, Melissa Hill, Caroline M. Vass, Jennifer Hammond, Sam Riedijk, Jasmijn E. Klapwijk, Eleanor Harding, Stina Lou, Ida Vogel, Lisa Hui, Charlotta Ingvoldstad‐Malmgren, Maria Johansson Soller, Kelly E. Ormond, Mahesh Choolani, Qian Zheng, Lyn S. Chitty, Celine Lewis

**Affiliations:** ^1^ Nuffield Department of Population Health Health Economics Research Centre University of Oxford Oxford UK; ^2^ National Institute for Health Research Oxford Biomedical Research Centre Oxford UK; ^3^ North Thames Genomic Laboratory Hub Great Ormond Street Hospital London UK; ^4^ Genetic and Genomic Medicine UCL Great Ormond Street Institute of Child Health London UK; ^5^ Manchester Centre for Health Economics The University of Manchester Manchester UK; ^6^ RTI Health Solutions Manchester UK; ^7^ Department of Clinical Genetics Erasmus MC Rotterdam The Netherlands; ^8^ BSc Paediatrics and Child Health The UCL Great Ormond Street Institute of Child Health London UK; ^9^ Center for Fetal Diagnostics Department of Clinical Medicine Aarhus University Aarhus Denmark; ^10^ DEFACTUM – Public Health & Health Services Research, Central Denmark Region Aarhus Denmark; ^11^ Department of Clinical Genetics Aarhus University Hospital Aarhus Denmark; ^12^ Reproductive Epidemiology Group Murdoch Children's Research Institute Parkville Victoria Australia; ^13^ Department of Perinatal Medicine Mercy Hospital for Women Heidelberg Victoria Australia; ^14^ Department of Obstetrics and Gynaecology Northern Health Epping Victoria Australia; ^15^ Center for Research and Bioethics Uppsala University Uppsala Sweden; ^16^ Center for Fetal Medicine Karolinska University Hospital Stockholm Sweden; ^17^ Department of Clinical Genetics Karolinska Hospital and Department of Molecular Medicine and Surgery Karolinska Institutet Stockholm Sweden; ^18^ Department of Genetics and Stanford Center for Biomedical Ethics Stanford University School of Medicine Stanford California USA; ^19^ Department of Health Sciences and Technology Health Ethics and Policy Lab ETH Zurich Zurich Switzerland; ^20^ Department of Obstetrics & Gynaecology National University Hospital Singapore Singapore; ^21^ Yong Loo Lin School of Medicine National University of Singapore Singapore Singapore; ^22^ Population, Policy and Practice UCL Great Ormond Street Institute of Child Health London UK

## Abstract

**Objective:**

We conducted a survey‐based discrete‐choice experiment (DCE) to understand the test features that drive women's preferences for prenatal genomic testing, and explore variation across countries.

**Methods:**

Five test attributes were identified as being important for decision‐making through a literature review, qualitative interviews and quantitative scoring exercise. Twelve scenarios were constructed in which respondents choose between two invasive tests or no test. Women from eight countries who delivered a baby in the previous 24 months completed a DCE presenting these scenarios. Choices were modeled using conditional logit regression analysis.

**Results:**

Surveys from 1239 women (Australia: *n* = 178; China: *n* = 179; Denmark: *n* = 88; Netherlands: *n* = 177; Singapore: *n* = 90; Sweden: *n* = 178; UK: *n* = 174; USA: *n* = 175) were analyzed. The key attribute affecting preferences was a test with the highest diagnostic yield (*p* < 0.01). Women preferred tests with short turnaround times (*p* < 0.01), and tests reporting variants of uncertain significance (VUS; *p* < 0.01) and secondary findings (SFs; *p* < 0.01). Several country‐specific differences were identified, including time to get a result, who explains the result, and the return of VUS and SFs.

**Conclusion:**

Most women want maximum information from prenatal genomic tests, but our findings highlight country‐based differences. Global consensus on how to return uncertain results is not necessarily realistic or desirable.

## Key points

**What's already known about this topic?**

- Prenatal genome‐wide sequencing approaches increase diagnostic yield but also the chance of identifying uncertain findings.
- It is unclear which test features drive women's preferences for prenatal genomic testing.

**What does this study add?**

- The findings add to our understanding of whether and how attitudes differ across countries with varying cultures and healthcare services.
- This work highlights the importance of guidelines tailored to individual countries as well as pre‐test counseling that identifies the personal values and preferences of patients.

## 1 INTRODUCTION

Ongoing innovations in prenatal diagnosis, such as chromosomal microarray analysis (CMA) and prenatal exome sequencing (ES), are increasing the possibility of finding a genetic diagnosis for the 2%–5% of pregnancies where a fetal anomaly has occurred.^1^, ^2^ Obtaining a genetic diagnosis following an abnormal ultrasound scan in pregnancy can have multiple clinical benefits: a diagnosis enables accurate counseling around prognosis; informs decision making about pregnancy management; guides delivery planning and perinatal management and facilitates reproductive autonomy and psychological preparation.^3^

Traditionally, prenatal diagnosis has relied on cytogenetic analysis, including karyotyping, and targeted genetic testing for suspected single gene disorders. Over the last decade, CMA has become a commonly used first‐line test, bringing higher diagnostic yields than karyotyping.^4^ More recently, prenatal testing options have widened further to include genome‐wide sequencing approaches such as ES and targeted panels which yield more diagnoses than either karyotyping or CMA alone.^5^, ^6^ CMA and ES facilitate a comprehensive analysis of the fetal genome, but as diagnostic yields increase so does the chance of findings that have prognostic uncertainties such as variants of uncertain significance (VUS), susceptibility loci with low penetrance, as well as known variants that are unrelated to the original reason for testing (secondary findings ‐ SFs) which may or may not be looked for (the latter sometimes called ‘incidental findings’), and which may increase an individual's risk for developing a condition but may not be 100% penetrant.

Several studies involving couples who have been offered CMA or ES during pregnancy have reported that they do want to receive uncertain results.^7^, ^8^, ^9^ Many couples, however, opt for prenatal testing because they want reassurance or definitive answers. However, uncertain findings can come as a surprise, with couples experiencing shock, anxiety and decision regret.^7^, ^8^, ^9^, ^10^, ^11^ Accordingly, understanding the preferences and priorities of women from around the globe for the types of prenatal tests that may reveal uncertain results is important and will help health professionals (HPs) to support parents when offering these tests. Notably, while HPs around the world clearly deal with similar sources of uncertainty, qualitative interviews with HPs from different countries have indicated variability in how this uncertainty is managed.^12^ In that study conducted with clinical scientists and clinicians (e.g. geneticists, genetic counselors) who conduct post‐test counseling around prenatal CMA and/or ES, five overarching sources of uncertainty were identified including: 1) incomplete knowledge for example, unclear pathogenicity and VUS; 2) unexpected findings for example, incidental findings; 3) uncertainty caused by the technology for example, technical validity of the result; 4) uncertainty related to the condition for example, conditions with incomplete penetrance; and 5) uncertainty related to clinical utility of the test that is, diagnostic yield. Interviews revealed that there was variation in reporting practices both between and across countries for VUS as well as who decides what results are reported.

To explore women's preferences for prenatal genomic tests that can reveal uncertain findings we have used a discrete‐choice experiment (DCE). DCEs are an established methodology used to elicit and quantify the preferences of stakeholders by asking them to choose between hypothetical options with differing attributes.^13^ DCE surveys delivered in healthcare settings present participants with a series of choice sets that feature particular attributes of an intervention that vary across a fixed number of clinically relevant levels. DCEs have been used widely in healthcare settings, including consideration of differing approaches to prenatal testing and screening.^14^, ^15^, ^16^, ^17^

The aim of this study was to understand the test features that drive women's preferences for prenatal genomic tests using a DCE administered in eight countries selected for diversity in both culture and healthcare system. A secondary aim was to explore the heterogeneity in these preferences both within and across countries.

## 2 METHODS

The design, administration, and analysis of the DCE survey followed good practice guidelines.^18^ Ethical approval for the study was granted by National Health Service (NHS) Health Research Authority London – Riverside. Research Ethics Committee reference: 18/LO/2120.

### 2.1 Development of discrete‐choice experiment choice scenarios

**FIGURE 1:**
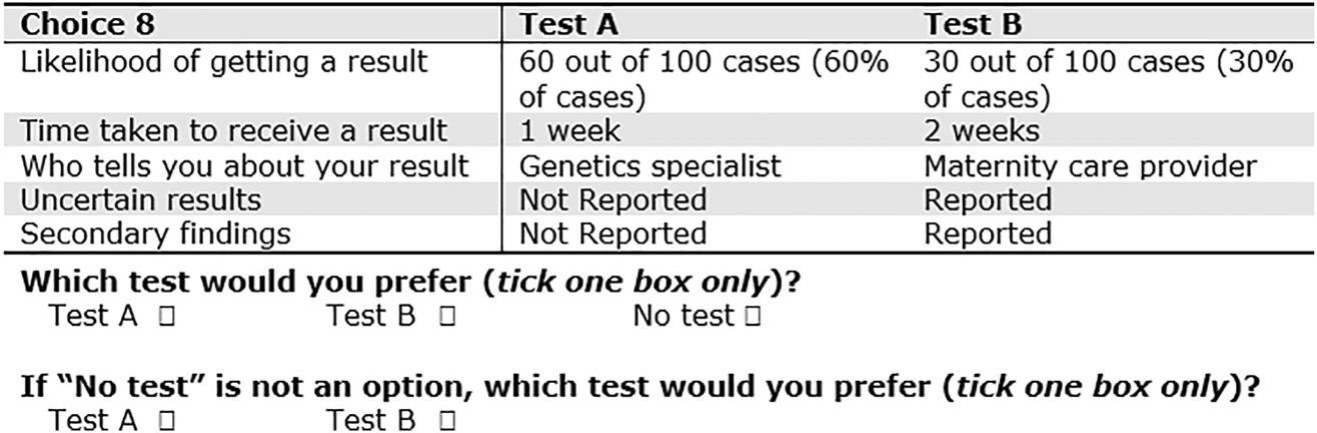
Example choice set

An extensive description of the development of the attributes and levels for the DCE survey is provided elsewhere.^19^ In brief, we applied a two‐phase mixed methods approach involving a systematic review of the literature, followed by semi‐structured interviews and a quantitative scoring exercise. This yielded five attributes (described in Table 1): diagnostic yield, reporting of variants of uncertain significance, reporting of SFs, time to receive results, and which healthcare provider explains the results. Each of these attributes had either two or three levels which were grounded in reality yet in some cases for example, diagnostic yield, represented the higher and lower ends of what was realistic to ‘encourage’ participants to make decisions and trade‐offs.^20^ An example of the sample choice questions is shown in Figure 1. In the DCE survey (supplementary information Figure 1), we first described a hypothetical scenario in which a couple attend their routine 20 weeks ultrasound scan and a fetal anomaly is suspected. The couple is subsequently offered invasive testing (presented as having a 0.5% risk of miscarriage – this risk estimate was chosen to strike a balance between how this risk is presented in different countries). Respondents were then presented with 13 choice questions, which is considered an acceptable number of choices to complete without introducing concerns around the impact of fatigue on responses.^21^ For each choice question, responders were asked to choose Test A, Test B or No Test. The No Test option was included to make the choice more realistic; in practice, women may choose not to have a test. This approach has been used in previous DCE studies looking at attributes of prenatal testing.^14^ Respondents were told that if they selected No Test, they would not get a diagnosis, waiting time would be zero, and no uncertain results or secondary findings would be reported. If they selected No Test, respondents were then asked which test they would prefer if No Test was not an option.

**TABLE 1:** List of attributes and their associated levels

Attribute	Levels
Likelihood of getting a result	5 out of every 100 cases (5% of cases)
	30 out of every 100 cases (30% of cases)
	60 out of every 100 cases (60% of cases)
Time taken to receive a result	1 week
	2 weeks
	4 weeks
Who explains your results to you	Genetic specialist with specialist knowledge of the test findings but who you have not met before
	Your main maternity care provider who you know well but who will not have specialist knowledge
Uncertain results	Uncertain results reported back to parents
	Uncertain results not reported back to parents
Secondary findings	Secondary findings reported back to parents
	Secondary findings not reported back to parents

The levels that were presented for Test A and Test B in each choice question were generated using an experimental design that was produced by Ngene (Choice Metrics 2018),^22^ specialist software for generating experimental designs for DCEs. Effects‐coding, where the variables for each level are replaced by −1, 0 or 1, was used for all attributes. The design included no constraints or interactions. For the initial design, a model averaging approach was applied with zero priors to generate multinomial logit (MNL) models with and without the opt‐out. To generate this design, following consultation within the study team we assumed that the opt‐out would be selected in 10% of the choice scenarios. A d‐efficient design was selected that exhibited level balance (each level appears an equal number of times) and had no level overlap (no repetition of attribute levels). Following a pilot (see below), this design was refined to include priors for all attribute levels, and to assume that the opt‐out would be selected in 8% of the choice scenarios (based on choices made in the pilot). The final design was again d‐efficient, with level balance and no level overlap.

### 2.2 Survey assembly

The final survey included: a) background information about prenatal testing, b) a description of the attributes and levels included in the DCE, c) an attribute ranking exercise, d) the DCE choice questions, e) a hypothetical scenario to ascertain preferences for targeted or broad genomic tests, f) the short form of the Intolerance of Uncertainty Scale,^23^ and g) questions to collect information on respondent characteristics (age, ethnicity, education, number of children, if they had a baby within the last 24 months, and when they had their last child) and maternity‐ and pregnancy‐related experiences. For the attribute ranking exercise, we included the attribute ‘test safety’, which we did not include in the DCE. Test safety has been found to be at the forefront of women's minds when they make decisions about prenatal tests.^14^ We therefore included it in the ranking exercise to check this previous finding. However, we decided not to include it as a DCE attribute as we were a) concerned that it would dominate women's decision‐making and would therefore override the importance of other attributes we were interested in, and b) ES cannot yet be delivered non‐invasively so it would have presented women an unrealistic test choice. One choice question was generated by the study team (separate to the experimental design) and inserted into the survey as question 6. This question was designed to be a ‘rationality check’; it included one ‘dominant’ choice alternative that was unequivocally the best choice that respondents could make given the levels presented for each attribute.

### 2.3 Sampling and data collection

An anonymous, online survey (supplementary information Figure 1) was administered to women from 8 countries: Australia, China, Denmark, Singapore, Sweden, the Netherlands, the UK and the USA through the international market research company Dynata (www.dynata.com). The survey was translated, where necessary, by bilingual members of the study team into Chinese, Danish, Dutch and Swedish, and was hosted through the online survey platform SurveyMonkey Inc (San Mateo, California, USA). Women aged 18–47 years who had given birth in the past 24 months were eligible to participate (an upper age‐limit of 45 was chosen as it was unlikely women would have had a child after this age. We increased this to 47 years to account for the fact women had to have had a child in the past 24 months to participate in the survey). Dynata circulated an email invitation to women on their market research panel who they believed to be within the eligible age range. The invitation described the topic of the survey (‘Genomics’), the approximate completion time (15 min) and the payment for completion (£1.25). Those who were interested in taking part clicked a link to begin the survey.

At the start of the survey, participants were provided with an information sheet about the study and were asked to tick a box indicating their consent prior to commencing the survey. Participants were then asked a series of eligibility questions (gender, had a baby in past 24 months, aged 18–47). Those who did not meet the inclusion criteria were screened out.

We aimed to collect a minimum number of 200 completed surveys (including both the pilot and main survey) for all countries except Denmark and Singapore where we aimed to collect 100 (due to the smaller number of eligible women available in these countries through Dynata). These numbers were chosen for practical reasons in terms of affordability and recruitment time. Quotas were set on education (no education through to upper secondary school education *v* higher education) and age of responder (18–32 and 33–47) so that there would be at least 50 women in each category in each country, other than in Denmark and Singapore where a minimum number of 30 women in each category was required.

### 2.4 Pilot and launch

To pilot the administration process, a ‘soft launch’ was conducted with 10% of the required sample (i.e. in countries where we were aiming for 200 completed surveys, in the pilot we evaluated how many survey invitations were required to be sent in order to collect 20 completed surveys). Recruitment rates were over 50% in each country for the pilot study (i.e. fewer than 40 survey invites were required in order to collect 20 completed surveys) indicating an adequate response rate and suggesting that the survey was straightforward to complete (median completion time 9 min). The pilot DCE data was analyzed by constructing an MNL model, and minor changes were made to the experimental design, as described above. Data collection for the main survey took place from 30^th^ July 2019 to 18^th^ August 2019.

### 2.5 Statistical analysis

Responses to the sociodemographic and experience‐related survey questions were analyzed using descriptive statistics. The choice data from the DCE were analyzed using a mixed logit (also called a random‐parameters logit) model that modeled test choice and the attribute levels of the hypothetical tests presented.^24^ In the mixed logit model, all parameters were assumed to be independent, random, and normally distributed, and the model was estimated using 500 Halton draws. All variables were effects‐coded to allow estimation of each attribute level given a mean effect of zero,^25^ and a constant term was included to model the choice of No Test (opting‐out). The coefficients generated by this model represent the relative preference weights for each attribute level included in the DCE.

The estimated preference weights were used to calculate the conditional relative importance for each attribute (the difference between the highest and lowest preference weights within each attribute) to show the importance in changes among the levels of one attribute compared with changes in other attributes.^26^ All statistical analyses were conducted in Stata 16.^27^

## 3 RESULTS

A total of 2190 participants clicked on the survey link. Of those, 951 were excluded (see Table 2) leaving 1239 participants included in the final analysis (57% response rate). Participant characteristics are summarized in Table 2, with additional details presented in supplementary information Table 1. The average (mean) age of respondents was 31. 4 years (median of 31 years), over a half (58.2%) had past experience undergoing screening for Down syndrome, and almost a quarter (23.6%) had experience of invasive testing. Over half (55.3%) had a higher education qualification, although this varied across countries (22.7% in Denmark compared to 86.6% in China). The final dataset can be found in the University College London (UCL) research data repository.^28^

**TABLE 2:** Summary of respondents' key characteristics

	Overall sample
	*N* = 1239
Age in years (mean)	31.4
Highest educational qualification	
No or elementary education	50 (4.0%)
Lower secondary school education	147 (11.9%)
Upper secondary school education	357 (28.8%)
Higher education	685 (55.3%)
Religious faith	
None	546 (44.1%)
Christian	435 (35.2%)
Jewish	24 (1.9%)
Muslim	78 (6.3%)
Hindu	25 (2.0%)
Buddhist	87 (7.0%)
Other	36 (2.9%)
Rather not answer	6 (0.5%)
Ever had down syndrome screening in a pregnancy	
Yes	721 (58.2%)
No	464 (37.4%)
Don't know	54 (4.4%)
Ever had invasive testing in any pregnancy	
Yes	292 (23.6%)
No	872 (70.4%)
Don't know	75 (6.1%)
Ever had test results in pregnancy that indicated that the baby had a genetic condition	
Yes	185 (14.9%)
No	1005 (81.1%)
Don't know	49 (4.0%)
Ever terminated a pregnancy because the baby had a health issue	
Yes	135 (10.9%)
No	1067 (86.1%)
Don't know	37 (3.0%)
Total children (mean)	1.8
Months since last baby was born (mean)	11.2

*Note*: Regarding response rate ‐ a total of 2190 participants clicked on the survey link. Of those, 951 were excluded because they did not consent (*n* = 95), dropped out immediately after consenting (*n* = 65), dropped out during the screening questions (*n* = 28), screened out as not eligible (*n* = 432), dropped out during the survey (*n* = 98), or completed the survey in under 4 min, indicating that they did not engage with the survey (*n* = 233). This left a final total of 1239 participant.

### 3.1 Ranking exercise

**FIGURE 2:**
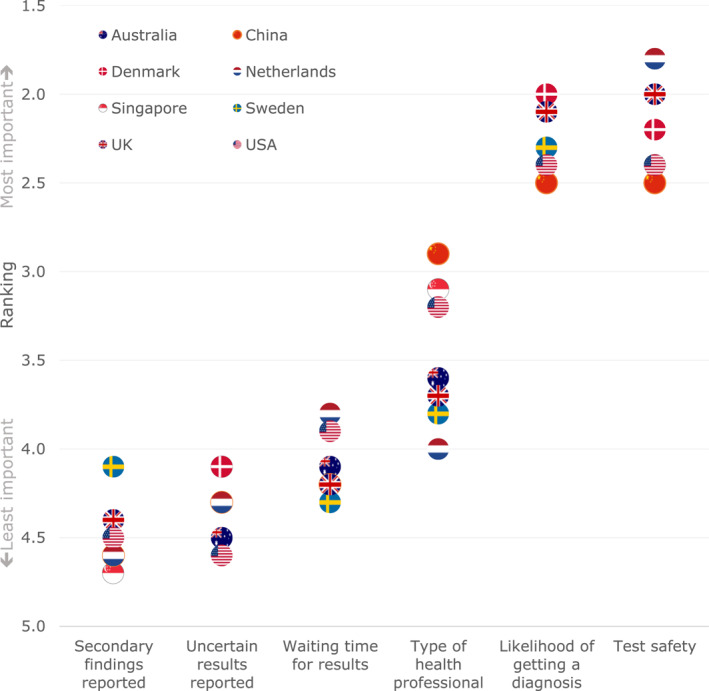
Ranking exercise

The results of the ranking exercise are presented in Figure 2 and detailed in supplementary information Table 2. Test safety was the most important attribute in all countries except Denmark, where the likelihood of getting a diagnosis was ranked most important. Reporting of SFs, uncertain results, and waiting time for results were the least important. Heterogeneity in the importance of the type of HP explaining the results was observed: this attribute was more important in China, Singapore and the USA, but less important in European countries.

### 3.2 Discrete‐choice experiment results

**FIGURE 3:**
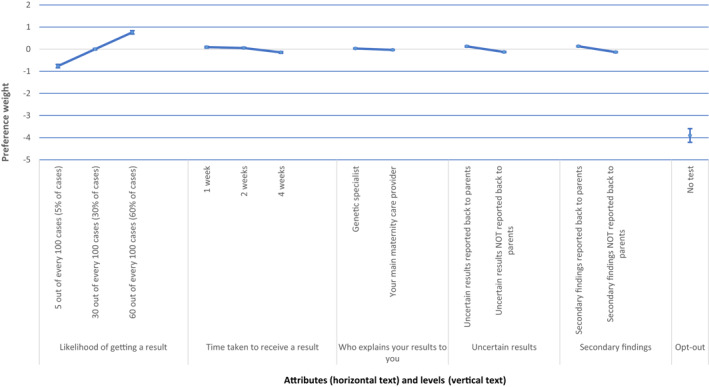
Plotted preference weights for the pooled sample (*n* = 1239). *Note*: The vertical bars showing the 95% confidence interval. In attributes where the bars do not overlap, the level was statistically different from the other (*p* < 0.05). More preferred levels have higher weights. For example, the preference weight for the 60%‐level of ‘likelihood of getting a result’ was greater than the 5%‐level, suggesting higher levels of this attribute were preferred. The vertical distance between the most and least‐preferred levels of an attribute illustrates the relative importance of the attribute, given the levels included in the study. In all study countries, the likelihood of getting a result was the most important attribute, and so that level is fixed

The preference‐weights for each attribute level are presented in Table 3 and plotted for the whole sample in Figure 3. The preference weights for each country are presented graphically in supplementary information Figures 2‐9. Considering the pooled results, women strongly favored prenatal testing over no testing (Table 3). As anticipated, women had a preference for tests with a higher likelihood of getting a result, shorter test turnaround times and preferred having uncertain results and SFs returned as opposed to not returned. For the time taken to receive a result, women preferred shorter waits compared with a 4‐week wait, but there was no statistically significant difference between 1‐week and 2‐week waits. Similarly, on average across the whole data set, women were indifferent between receiving results from a genetics specialist or their main maternity care provider. For the rationality check question, the majority of participants (71.8%) gave the expected answer, with 8.2% opting out of testing.

**TABLE 3:** Estimated coefficients for each country and the overall sample (*n* = 1239)

	Australia	China	Denmark	Netherlands	Singapore	Sweden	UK	USA	ALL
	*N* = 178	*N* = 179	*N* = 88	*N* = 177	*N* = 90	*N* = 178	*N* = 174	*N* = 175	*N* = 1239
5 out of every 100 cases (5% of cases)	−0.807*** (0.106)	−0.162** (0.055)	−1.274*** (0.218)	−1.131*** (0.122)	−1.289*** (0.235)	−0.888*** (0.135)	−0.969*** (0.112)	−0.616*** (0.085)	−0.763*** (0.039)
30 out of every 100 cases (30% of cases)	−0.001 (0.045)	0.025 (0.038)	0.04 (0.072)	0.043 (0.048)	−0.013 (0.092)	−0.004 (0.046)	−0.053 (0.044)	−0.01 (0.042)	0.002 (0.017)
60 out of every 100 cases (60% of cases)	0.808*** (0.105)	0.137** (0.056)	1.235*** (0.216)	1.088*** (0.12)	1.302*** (0.237)	0.892*** (0.134)	1.022*** (0.111)	0.625*** (0.085)	0.0762*** (0.039)
1 week	0.132** (0.055)	0.051 (0.039)	0.206** (0.098)	0.005 (0.058)	0.041 (0.09)	0.155** (0.055)	0.115** (0.049)	0.081* (0.047)	0.091*** (0.019)
2 weeks	0.138** (0.047)	−0.017 (0.038)	0.202** (0.078)	0.125** (0.051)	0.074 (0.078)	0.052 (0.049)	−0.011 (0.047)	0.036 (0.044)	0.055*** (0.017)
4 weeks	−0.27*** (0.055)	−0.035 (0.038)	−0.408*** (0.102)	−0.13** (0.058)	−0.115 (0.089)	−0.207*** (0.055)	−0.104** (0.049)	−0.117** (0.046)	−0.146*** (0.019)
Genetic specialist with specialist knowledge of the test findings but who you have not met before	0.06* (0.033)	0.062** (0.028)	−0.129** (0.059)	−0.026 (0.038)	0.064 (0.062)	0.074** (0.035)	0.039 (0.032)	0.016 (0.031)	0.032** (0.012)
Your main maternity care provider who you know well but who will not have specialist knowledge	−0.06* (0.033)	−0.062** (0.028)	0.129** (0.059)	0.026 (0.038)	−0.064 (0.062)	−0.074** (0.035)	−0.039 (0.032)	−0.016 (0.031)	−0.032** (0.012)
Uncertain results reported back to parents	0.117** (0.038)	0.096*** (0.027)	0.102* (0.057)	0.146** (0.046)	0.315*** (0.075)	0.149*** (0.039)	0.113** (0.039)	0.097** (0.035)	0.0127*** (0.014)
Uncertain results not reported back to parents	−0.117** (0.038)	−0.096*** (0.027)	−0.102* (0.057)	−0.146** (0.046)	−0.315*** (0.075)	−0.149*** (0.039)	−0.113** (0.039)	−0.097** (0.035)	−0.0127*** (0.014)
Secondary findings reported back to parents	0.173*** (0.036)	0.099*** (0.024)	0.158** (0.077)	0.123** (0.042)	0.242*** (0.062)	0.188*** (0.041)	0.193*** (0.032)	0.019 (0.031)	0.132*** (0.013)
Secondary findings not reported back to parents	−0.173*** (0.036)	−0.099*** (0.024)	−0.158** (0.077)	−0.123** (0.042)	−0.242*** (0.062)	−0.188*** (0.041)	−0.193*** (0.032)	−0.019 (0.031)	−0.132*** (0.013)
Alternative specific constant (opt‐out)	−3.488*** (0.394)	−4.945*** (0.649)	−5.303*** (0.88)	−3.627*** (0.415)	−3.876*** (0.65)	−3.712*** (0.385)	−3.619*** (0.385)	−2.857*** (0.269)	−3.902*** (0.158)
Log likelihood (LL)	−1577.477	−1532.053	−648.733	−1472.284	−732.513	−1496.441	−1493.263	−1667.733	−10817.339

*Note*: Standard errors in parentheses. The log likelihood is included for completeness in the table, but they are relative values and can only be used to compare goodness of fit across models within the same country and do not have a meaning in absolute terms. The constant term indicates the average effect of all factors that influence opt‐out choices that are not explained by attributes in the DCE, its sign and significance suggests a propensity to opt‐in to screening. Standard deviations are presented in Supplementary Table 3.

*= *p* < 0.1; **= *p* < 0.05; ***= *p* < 0.01.

Information on the difference between preference weights for different attribute levels can be used to quantify the relative importance of moving between levels across different attributes. For example, the difference in preference weights when the likelihood of a result improves from 30% to 60% is approximately 0.075, whereas the difference in preference weights when moving from a turnaround time of 4‐week to 2‐week is approximately 0.1 per week. Women are therefore willing to wait an additional 5 days for test results if the likelihood of getting a result increases by 30%.

### 3.3 Conditional relative importance

**FIGURE 4:**
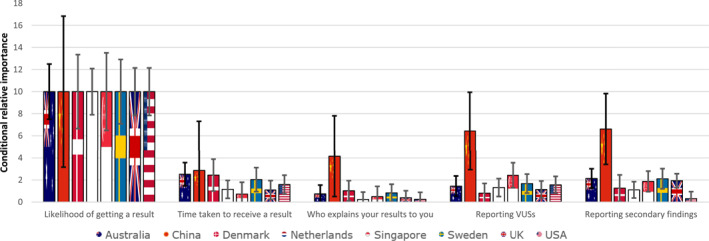
Relative importance of each test feature by country. *Note*: The vertical bars show the 95% confidence interval

Figure 4 shows the relative importance of each test attribute by country. Across countries, there was notable heterogeneity in preferences. For example, in Australia, Denmark and the US the second most important attribute was the time taken to receive a result, whereas this attribute was of little‐to‐no importance in China and Singapore. In China, Sweden, and the UK the second most important attribute was the opportunity to receive SFs, but in the US this result was of little‐to‐no importance. In Singapore and the Netherlands, the second most important attribute was the reporting of uncertain results, whereas in Denmark this attribute was of little‐to‐no importance. Who explains your results was of low or no importance relative to the other attributes in Australia, Sweden, Netherlands, Singapore, the UK and the US; however, it was important in China and Denmark. There was a differences in preferences around who explains your results (Table 3); in China and Sweden, the genetic specialist was preferred, whereas in Denmark the main maternity care provider was preferred.

### 3.4 Preferences for no testing

Overall, participants selected an invasive test (Test A or B) in 93% of all choices (i.e. ‘no test’ was selected in 7% of choices), and 32% of women (*n* = 397) opted out at least once. The results of the logistic regression (Table 4) indicate that women who opted out at least once were less likely to have experience undergoing either Down syndrome screening or invasive testing. Women who had higher education were more likely to always opt in to testing (compared to those with secondary school or no education). The other covariates investigated (age, number of children, religion, time since last baby, terminating a pregnancy or having received a test result which showed a genetic condition, intolerance for uncertainty) were uncorrelated.

**TABLE 4:** Logit model of stated preferences for no test

	Estimate	
Age	0.004	(0.01)
Number of children	0.113	(0.07)
Time since last baby	0.008	(0.01)
Base case (no education)		
Lower secondary school education	−0.338	(0.35)
Upper secondary school education	−0.510	(0.32)
Higher education	−0.662*	(0.32)
Base case (none)		
Christian	0.264	(0.14)
Jewish	0.831	(0.45)
Muslim	0.076	(0.27)
Hindu	0.026	(0.47)
Buddhist	0.290	(0.27)
Other	−0.124	(0.40)
Rather not answer	0.312	(0.90)
Base case (had down syndrome screening)		
No	0.387**	(0.14)
Don't know	0.560	(0.34)
Base case (had invasive testing)		
No	0.695***	(0.19)
Don't know	0.853*	(0.33)
Base case (test indicated baby had genetic condition)		
No	0.188	(0.23)
Don't know	0.688	(0.44)
Base case (terminated pregnancy)		
No	0.012	(0.27)
Don't know	0.609	(0.51)
Intolerance for uncertainty	−0.011	(0.01)
Constant	−1.310*	(0.56)
N	1232	

*Note*: Standard errors in parentheses. Negative (positive) coefficients imply variable is associated with a reduction (increase) in the odds of opting‐out.

*p < 0.05; **p < 0.01; ***p < 0.001.

### 3.5 Preferences for targeted or broad genomic tests

In the pooled sample, and in all individual countries except China, women preferred broad rather than targeted tests (49% v 28% respectively; supplementary information Table 1). In China, women preferred targeted tests rather than broad tests (51% v 43% respectively). Regarding who should make the decision about which test to have (broad or targeted), across all countries, women would want to make the decision themselves/with their partners.

## 4 DISCUSSION

We used a DCE survey to explore the preferences of women from eight socially and ethnically diverse countries for prenatal genomic tests that can reveal uncertain findings. As far as we know, this is the first DCE to explore women's preferences for uncertain prenatal results with an international cohort. The findings are therefore useful as they add to our understanding of whether and how attitudes differ across countries with varying cultures and healthcare services. The ranking exercise indicated that test safety was the most important feature of prenatal tests and around one‐third of women opted out of testing in at least one choice scenario, underscoring that safety is at the forefront of women's minds when considering prenatal tests, a finding that has been reported elsewhere.^14^ Across all countries, the likelihood of receiving a result was the most important attribute, with women prepared to wait longer for results if a test could provide a higher diagnostic yield. Women also preferred tests with a higher likelihood of getting a result, shorter turnaround times and tests where VUS and SFs were reported, although return of VUS and SFs were not found to be important in the ranking exercise.

Women expressed a preference for prenatally receiving uncertain findings such as VUS and SFs. Similar findings regarding the return of VUS have been reported in other hypothetical studies with parents,^29^, ^30^ although the experiences of women who have received prenatal VUS show that this can cause anxiety, watchful waiting, decisional‐regret and feeling overwhelmed.^9^, ^10^, ^11^, ^31^, ^32^, ^33^, ^34^ Professionals tend to be more conservative about reporting uncertain findings in prenatal settings,^35^, ^36^, ^37^ and guidelines^38^ as well as approaches vary.^39^, ^40^, ^41^, ^42^ With regards to SFs, there is ongoing debate around whether it is appropriate to return SFs for babies and children as this would prevent them from making their own autonomous decision about adult‐onset disorders and carrier status.^43^, ^44^ Published recommendations^45^, ^46^ reflect the consensus that medically‐actionable SFs should be routinely offered, with European guidelines more cautious than those from the United States.^47^ Research reporting parent choices about SF in a prenatal setting is limited, with one recent study from the US finding that 86% (249/289) of parents chose to receive SFs when offered prenatal ES.^48^ Whilst our study concerns theoretical behavior and it could be hypothesized that this does not routinely map on to actual behavior, this study does in fact support our hypothesized findings. Moreover, at least within the preference literature, there is some evidence that stated preferences can match “revealed” actions in the real world.^49^

Several country‐specific differences in women's preferences for receiving uncertain results were identified, including length of time to get a result, who explains the result, and the return of VUS and SFs. Differences in the importance of how long it takes to get a result may reflect national legislation around termination of pregnancy, which varies in terms of time limits, mandatory counseling and third party authorization procedures.^50^ These differences may also be related to religious, ethical and cultural values concerning the acceptability of terminating a pregnancy and the moral status of the fetus,^51^, ^52^ as well as whether government benefits, health services and/or support groups exist for children with disabilities^53^ and whether shame or stigma is associated with the birth of a baby with a genetic condition.^54^ Varying attitudes towards the importance of receiving SFs and VUS may also reflect differing views around ownership of genomic data^55^ and whether the costs of prenatal tests are covered by state or out‐of‐pocket expenses^56^; in a recent international study, some HPs felt that they had a responsibility to return VUS when patients had paid out‐of‐pocket.^39^ Differences in preferences towards who explains the test results may be related to whether or not there is easy access to genetic services,^57^ genetic health literacy^58^, ^59^ or the role played by midwives in different countries.^60^ Overall, our findings support the development of guidance around return of uncertain results that take into account cultural and health system differences. These differences highlight the importance of tailored counseling, during which prospective parents can articulate their values, and identify and discuss options.

Our findings have several implications for clinical practice. First, given that diagnostic yield was at the forefront of women's minds, HPs should ensure that they explain to patients that they may not get a diagnostic result from ES, and that diagnostic yield can be dependent on factors such as fetal phenotype and approach to analysis and reporting. Providers should also be transparent about the expected diagnostic yield of the different test options and why. Second, given women's preference to receive VUS and SFs, pre‐test counseling should include a discussion of whether or not these, as well as other uncertain results such as susceptibility‐loci, will be reported. Third, if VUS are going to be reported, the current process for reanalysis of VUS should be discussed with parents as new published evidence or additional phenotypic information following birth can result in reclassification. Finally, this study shows that women can and will make trade‐offs between the different test features, including turnaround time and the range of results they can receive. This underscores the importance of genetic counseling services supporting parents in making decisions that fit with their values, and helping them to meet as many of their decisional goals as possible.^61^

### 4.1 Study strengths and limitations

A key strength of our study is that it is based on attributes identified through a rigorous mixed‐methods approach and included a large and diverse sample. Our study also has several limitations. First, the preference‐weights for attribute levels are conditional on the ranges of levels included in the DCE. This could impact the relative importance of different attribute levels. For example, presenting a smaller range for the likelihood of a test result could reduce the importance of that attribute relative to others. Furthermore, the preference‐weights are contingent on the attributes presented to respondents, and other test features may influence women's preferences in practice. Second, test safety was identified as the most important attribute in the ranking exercise. However, although we state the risk of miscarriage associated with invasive testing in our survey, we did not state the risk of miscarriage in the ‘no testing’ scenario. This may have led respondents to underestimate this risk for no testing when making their choices. We also stated the risk as 0.5% to strike a balance between how this risk is presented in different countries, however, the risk has been calculated to be lower than this in a recent meta‐analysis (0.3% for amniocentesis and 0.2% for chorionic villus sampling).^62^ Whilst we presented risk as both a frequency and a percentage, we did not present it pictorially which is considered good practice. Third, the study sample has not been weighted to accurately reflect each countries' particular population, and may therefore not reflect the preferences of the broader population of women seeking prenatal testing in the countries studied. Finally, due to resource limitations, only women who had had a baby were included in this study. Fathers and pregnant women may have different preferences.

## 5 CONCLUSION

The results of this study indicate that most women want to receive maximum information from prenatal genomic testing. However, country‐based differences do exist, highlighting the importance of pre‐test counseling that identifies the personal values and preferences of patients, as well as guidelines tailored to individual countries. Prenatal ES is set to have a significant impact on parental decision‐making following the identification of an abnormal ultrasound. Whilst this study provides much ‘food‐for‐thought’, there is still much to learn about the impact of these more advanced tests on decision‐making and patient experience. Further qualitative research should be undertaken to understand why women have a preference for maximum information from genomic tests in this context, and to establish if different stakeholders (e.g. pregnant women, men, HPs) have different preferences for these tests.

## CONFLICT OF INTEREST

James Buchanan received travel expense reimbursement from Illumina to attend meetings.

## Supporting information

Supplementary Material 1

Supplementary Material 2

Table S1

Table S2

Table S3

## Data Availability

The dataset from this work can be accessed through the UCL Data Repository: https://doi.org/10.5522/04/18692549.
