# Supplementary material for: Factors that impact on women's decision‐making around prenatal genomic tests: An international discrete choice survey
Source: Prenat Diagn. 2022 Apr 30;42(7):934–46. doi: 10.1002/pd.6159 (PMC9325352; doi:10.1002/pd.6159)
Supplement: Supplementary file 1 — Supplementary Material 1 [file PD-42-934-s005.pdf]

Great Ormond Street Hospital for Children NHS Foundation Trust

wellcome trust

What aspects of genetic testing in pregnancy do parents value most?

A survey study

What is the purpose of the study?

We are inviting you to complete a survey for our research study. The study is being conducted so that we can understand more about how people make decisions regarding new prenatal tests in pregnancy. Before you decide, we would like you to understand what the study involves. Please take time to read the following information carefully.

Who is organising and funding the research?

This research is organised by the Research Team at Great Ormond Street Hospital and UCL Institute of Child Health, London, UK. The study is funded by a Small Grant in Humanities and Social Science from the Wellcome Trust, a research charity based in London, UK**.** Great Ormond Street Hospital is the sponsor for this study based in England. Great Ormond Street Hospital will be using information from you in order to undertake this study and will act as the data controller for this study. This means that we are responsible for looking after the information from this study and using it properly. Great Ormond Street Hospital will keep non-identifiable information about you (e.g. age, sex, ethnicity) for 3 years after the study has finished.

What will happen to my data, and how will it be used?

The only people that will have access to the data from this survey will be researchers in Great Ormond Street Hospital and Oxford University. The people who analyse the information will not be able to identify you and will not be able to find out your name, or contact details. The findings from this work will be written up as a publication, but no names or details will be used.

Your rights to access, change or move your information are limited, as we need to manage your information in specific ways in order for the research to be reliable and accurate. If you withdraw from the study, we will keep the information about you that we have already obtained. To safeguard your rights, we will use the minimum personally-identifiable information possible. You can find out more about how we use your information at **{** HYPERLINK "https://www.gosh.nhs.uk/our-research/our-research-infrastructure/joint-rd-office/gdpr-and-research" **}** and/or by contacting our Data Protection Officer at **{** HYPERLINK "mailto:your.data@gosh.nhs.uk" **}**.

Who has reviewed the study?

This research has been looked at by an independent group of people, called a Research Ethics Committee, to protect your interests. This study has been reviewed and given favourable opinion by the London - Riverside Committee.

Do I have to take part?

No. Participation is voluntary and you can change your mind about being in the study at any time, without giving a reason.

How long will the survey take?

The survey should take no longer than 15 minutes to complete.

Where and how will my survey data be stored?

All survey data will be stored securely at Great Ormond Street Hospital and Oxford University on a password protected computer.

Who do I contact if I have any questions?

If you have any questions, please contact the research team. Our contact details are: Celine Lewis **{** HYPERLINK "mailto:celine.lewis@ucl.ac.uk" **}**

SOME QUESTIONS ABOUT YOU

1. What is your age?: …………………………………...

2. What is your sex?
   Male ☐
   Female ☐
   Intersex ☐

3. **Have you had a baby in the last 24 months?**

☐ Yes ☐ No

4. **How many children do you have?**
   ☐ I have had ………………………child(ren) ☐ No children

5. **What is your highest educational qualification?**
   *Please tick **one** box*

No education/Early childhood education/ Primary school (up to 11 years)/ Elementary school education
Lower secondary school education (up to 16 years) (GCSEs, O levels or equivalent)
Upper secondary school education (up to 18 years) (A levels of equivalent)
Higher education, university degree or equivalent and above

6. **Do you have a religious faith?**

   None
   Christian (any form) ☐ Jewish ☐
   Buddhist ☐ Muslim ☐
   Hindu ☐ Other ☐

7. **How religious are you?**

Not very religious ☐
Quite religious ☐
Very religious ☐

8. **Which of the following best describes your ethnicity?**
   *Please tick **one** box*

   White/White British ☐
   Asian/Asian British ☐
   Black/Black British ☐
   Mixed ☐
   Other ☐

WOMEN'S VIEWS OF PRENATAL TESTS FOR GENETIC CONDITIONS DURING PREGNANCY

BACKGROUND

Discovering that the baby is not developing as expected can occur in around 2-5% of pregnancies. Sometimes this happens during a routine ultrasound scan. Parents may be offered an invasive test. This might be an amniocentesis or CVS test that checks the baby's DNA. By looking at the baby's DNA, we can look for changes that may cause genetic conditions. Down syndrome is one of the most common genetic conditions that can be diagnosed during pregnancy. But there are many genetic conditions that are much rarer. These could impact the health and/or development of the baby.

Benefits of prenatal testing can include:

- A diagnosis
- Information about the condition and its impact on the health and/or development of the baby.
- Helping parents to plan for the birth.
- Making decisions about whether or not to continue with the pregnancy.

There is still a lot we don't understand about our DNA. It can be difficult to know whether some of the changes that we find in the baby's DNA are part of normal human differences, or whether they will have an impact on the health of the baby.

Some parents want as much information as possible about the health of their baby during pregnancy even if some of that information may be uncertain. Others prefer not to receive uncertain information during their pregnancy. The aim of this study is to understand what *you* think about uncertain test results during pregnancy.

In this survey you will be presented with a number of **imaginary situations** in which different tests are described. You will be asked to choose which test you prefer out of a number of different options. **There are no right or wrong answers**. We are just interested in your views.

Please read the list of factors below. As you read these think about which of these would be most important to you when considering a test in pregnancy.

- **Likelihood of getting a result:**
  - Tests can vary in how likely you will get a result that explains the cause of the baby's health issue.
  - For the tests described here a result will be found in 5 out of every 100 cases (**5% of cases**), 30 out of every 100 cases (**30% of cases**) or 60 out of every 100 cases (**60% of cases**)
  - If you do not choose a test you will not get a diagnosis. i.e. The likelihood of getting a diagnosis is 0 out of every 100 cases (0% of cases)

- **Time taken to receive a result:**
  - Tests can vary in how long it takes to get a result.
  - With the tests described here you could get your results in **1 week**, **2 weeks** or **4 weeks**.
  - If you do not choose a test, you will not get a result. i.e. There is no waiting time for results

- **Who explains your results to you:**
  - The health professional who explains your results can vary.
  - This could be a **genetics specialist** with specialist knowledge of the test findings but who you have not met before.
  - Or it could be your main **maternity care provider** who you know well but who will not have specialist knowledge.
  - If you do not choose a test, you will not get a result. i.e. no-one will explain your results to you.

- **Uncertain results:**
  - Sometimes, we find changes in the DNA but don't know if they are causing the health issue in the baby.
  - It can be hard to predict what the impact of these changes will be for the baby.
  - These uncertain results are either **reported** back to parents, or are **not reported** back to parents.
  - If you do not choose a test, you will not get any uncertainty result. i.e. no uncertain results are reported back to parents.

- **Secondary findings:**
  - We may find changes in the baby's DNA which cause diseases not related to the health issue in the baby. These might have health implications for the baby and possibly for the parents in later life.
  - These are known as 'secondary findings'. They may increase the chance of developing cancer or heart disease. These can often be treated or prevented.
  - These secondary findings are either **reported** to parents or are **not reported** to parents.
  - Some people want to receive secondary findings. They can act on the information. For example, they can go for more regular check-ups. Other people would rather not know. It may cause unnecessary worry about the illness which may not occur at all.
  - If you do not choose a test, you will not get any secondary findings. i.e. no secondary findings are reported back to parents.

SECTION A: The following questions ask what features of prenatal tests are most important to you

1. **Please rank the following test features in order of importance to you.**
   Put a 1 in the box for the most important; a 2 for the second most important; a 3 for the third most important; a 4 for the fourth most important and a 5 for the fifth most important, 6 for the least important.

{ FORMCHECKBOX } Test with the greatest likelihood of getting a result

{ FORMCHECKBOX } Type of health professional who explains the test result to you

{ FORMCHECKBOX } Test that is as safe as possible, with the lowest possible risk of a miscarriage

{ FORMCHECKBOX } Test with the shortest waiting time for results

{ FORMCHECKBOX } Test that reports secondary findings

{ FORMCHECKBOX } Test where all DNA changes are reported, even those where the impact on the health of the baby is unclear

SECTION B: The following questions ask you to compare two prenatal tests and indicate which one you would choose.

Consider the following situation: *A pregnant woman goes for her **routine 20 week ultrasound scan** at the hospital to check the development of the baby. During the appointment, something is seen on the scan. This may indicate that the baby has a genetic condition. This may impact on the baby's health and/or development. The couple are offered **invasive testing** to try to find out if the baby has a genetic condition. Invasive tests have a small risk of miscarriage (around 0.5*%, or around 1 in 200)

There are 13 choices listed below. Each choice involves **two different tests, which are slightly different in small ways**. **However, both tests are invasive.**

Imagine you are this woman and tick the box to show which option you would prefer; A, B or No test if you would choose not to have an invasive test. There are no right or wrong answers – we are just interested in your views.

**Choice 1**	**Test A**	**Test B**
Likelihood of getting a result	30 out of 100 cases (30% of cases)	60 out of 100 cases (60% of cases)
Time taken to receive a result	1 week	4 weeks
Who tells you about your result	Genetics specialist	Maternity care provider
Uncertain results	Not Reported	Reported
Secondary findings	Reported	Not Reported

Which test would you prefer (*tick one box only*)?
Test A ☐ Test B ☐ No test ☐

If "No test" is not an option, which test would you prefer (*tick one box only*)?
Test A ☐ Test B ☐

**Choice 2**	**Test A**	**Test B**
Likelihood of getting a result	30 out of 100 cases (30% of cases)	5 out of 100 cases (5% of cases)
Time taken to receive a result	1 week	4 weeks
Who tells you about your result	Maternity care provider	Genetics specialist
Uncertain results	Not reported	Reported
Secondary findings	Not Reported	Reported

Which test would you prefer (*tick one box only*)?
Test A ☐ Test B ☐ No test ☐

If "No test" is not an option, which test would you prefer (*tick one box only*)?
Test A ☐ Test B ☐

**Choice 3**	**Test A**	**Test B**
Likelihood of getting a result	5 out of 100 cases (5% of cases)	60 out of 100 cases (60% of cases)
Time taken to receive a result	4 weeks	1 week
Who tells you about your result	Genetics specialist	Maternity care provider
Uncertain results	Not reported	Reported
Secondary findings	Reported	Not Reported

Which test would you prefer (*tick one box only*)?
Test A ☐ Test B ☐ No test ☐

If "No test" is not an option, which test would you prefer (*tick one box only*)?
Test A ☐ Test B ☐

Choice 4	Test A	Test B
Likelihood of getting a result	60 out of 100 cases (60% of cases)	5 out of 100 cases (5% of cases)
Time taken to receive a result	2 weeks	1 week
Who tells you about your result	Maternity care provider	Genetics specialist
Uncertain results	Not reported	Reported
Secondary findings	Reported	Not reported

Which test would you prefer (*tick one box only*)?

Test A ☐ Test B ☐ No test ☐

If "No test" is not an option, which test would you prefer (*tick one box only*)?

Test A ☐ Test B ☐

Choice 5	Test A	Test B
Likelihood of getting a result	5 out of 100 cases (5% of cases)	30 out of 100 cases (30% of cases)
Time taken to receive a result	2 weeks	4 weeks
Who tells you about your result	Genetics specialist	Maternity care provider
Uncertain results	Reported	Not Reported
Secondary findings	Reported	Not Reported

Which test would you prefer (*tick one box only*)?

Test A ☐ Test B ☐ No test ☐

If "No test" is not an option, which test would you prefer (*tick one box only*)?

Test A ☐ Test B ☐

Choice 6*	Test A	Test B
Likelihood of getting a result	60 out of 100 cases (60% of cases)	5 out of 100 cases (5% of cases)
Time taken to receive a result	1 week	4 weeks
Who tells you about your result	Genetics specialist	Genetics specialist
Uncertain results	Not Reported	Not reported
Secondary findings	Not Reported	Not Reported

Which test would you prefer (*tick one box only*)?

Test A ☐ Test B ☐ No test ☐

If "No test" is not an option, which test would you prefer (*tick one box only*)?

Test A ☐ Test B ☐

Choice 7	Test A	Test B
Likelihood of getting a result	5 out of 100 cases (5% of cases)	30 out of 100 cases (30% of cases)
Time taken to receive a result	1 week	2 weeks
Who tells you about your result	Maternity care provider	Genetics specialist
Uncertain results	Reported	Not Reported
Secondary findings	Reported	Not Reported

Which test would you prefer (*tick one box only*)?

Test A ☐ Test B ☐ No test ☐

If "No test" is not an option, which test would you prefer (*tick one box only*)?

Test A ☐ Test B ☐

Choice 8	Test A	Test B
Likelihood of getting a result	60 out of 100 cases (60% of cases)	30 out of 100 cases (30% of cases)
Time taken to receive a result	1 week	2 weeks
Who tells you about your result	Genetics specialist	Maternity care provider
Uncertain results	Not Reported	Reported
Secondary findings	Not Reported	Reported

Which test would you prefer (*tick one box only*)?

Test A □ Test B □ No test □

If "No test" is not an option, which test would you prefer (*tick one box only*)?

Test A □ Test B □

Choice 9	Test A	Test B
Likelihood of getting a result	60 out of 100 cases (60% of cases)	5 out of 100 cases (5% of cases)
Time taken to receive a result	2 weeks	1 week
Who tells you about your result	Genetics specialist	Maternity care provider
Uncertain results	Reported	Not Reported
Secondary findings	Not Reported	Reported

Which test would you prefer (*tick one box only*)?

Test A □ Test B □ No test □

If "No test" is not an option, which test would you prefer (*tick one box only*)?

Test A □ Test B □

Choice 10	Test A	Test B
Likelihood of getting a result	5 out of 100 cases (5% of cases)	60 out of 100 cases (60% of cases)
Time taken to receive a result	2 weeks	4 weeks
Who tells you about your result	Maternity care provider	Genetics specialist
Uncertain results	Reported	Not reported
Secondary findings	Not Reported	Reported

Which test would you prefer (*tick one box only*)?

Test A □ Test B □ No test □

If "No test" is not an option, which test would you prefer (*tick one box only*)?

Test A □ Test B □

Choice 11	Test A	Test B
Likelihood of getting a result	60 out of 100 cases (60% of cases)	30 out of 100 cases (30% of cases)
Time taken to receive a result	4 weeks	1 week
Who tells you about your result	Maternity care provider	Genetics specialist
Uncertain results	Not Reported	Reported
Secondary findings	Not Reported	Reported

Which test would you prefer (*tick one box only*)?

Test A □ Test B □ No test □

If "No test" is not an option, which test would you prefer (*tick one box only*)?

Test A □ Test B □

Choice 12	Test A	Test B
Likelihood of getting a result	30 out of 100 cases (30% of cases)	5 out of 100 cases (5% of cases)
Time taken to receive a result	4 weeks	2 weeks
Who tells you about your result	Maternity care provider	Genetics specialist
Uncertain results	Reported	Not Reported
Secondary findings	Reported	Not Reported

Which test would you prefer (*tick one box only*)?
Test A ☐ Test B ☐ No test ☐

If "No test" is not an option, which test would you prefer (*tick one box only*)?
Test A ☐ Test B ☐

Choice 13	Test A	Test B
Likelihood of getting a result	30 out of 100 cases (30% of cases)	60 out of 100 cases (60% of cases)
Time taken to receive a result	4 weeks	2 weeks
Who tells you about your result	Genetics specialist	Maternity care provider
Uncertain results	Reported	Not Reported
Secondary findings	Not Reported	Reported

Which test would you prefer (*tick one box only*)?
Test A ☐ Test B ☐ No test ☐

If "No test" is not an option, which test would you prefer (*tick one box only*)?
Test A ☐ Test B ☐

SECTION C: A HYPOTHETICAL SCENARIO

Consider the following situation
A pregnant woman goes for her 12 week Down's syndrome screening test. She finds out there is an increased chance that the baby has Down's syndrome. She decides to have invasive testing to know for certain. She is offered two types of tests. Both are equally reliable:

Test 1: *This test is a* ***targeted test.*** *It will only provide information about Down's syndrome and two other serious conditions. These are called Edward's syndrome and Patau's syndrome.*

- *Down's syndrome causes some level of learning disability. This can vary from mild to severe. Some people will be more independent and do things like get a job. Other people might need more regular care. People with Down's syndrome may be more likely to have other health conditions. This includes heart conditions, and problems with the digestive system, hearing and vision. Sometimes these can be serious. But many can be treated.*

- *Nearly all babies with Edwards' syndrome or Patau's syndrome will die before or shortly after birth. Some babies may survive to adulthood, but this is rare.*

Test 2: *This is* ***a broader test.*** *It will provide* ***the same information as Test 1.*** *It will also look for* ***other conditions*** *that are known to affect the baby's health.*

- *Some changes found increase the baby's chance of having autism, learning disability or epilepsy.*

- *These conditions have what we call variable expression. Some people have no symptoms at all. Others will have severe symptoms. Unfortunately, there is no way of knowing in each case.*
    - *For example, it may identify that the baby has a condition called DiGeorge syndrome. DiGeorge syndrome is a condition present from birth that can cause a range of lifelong problems, including heart defects and learning difficulties (including delays in learning to walk or talk, or autism). The severity of the condition varies. Some children can be severely ill, but many others may grow up without realising they have it.*

- *This test will also reveal uncertain results. These are changes in the DNA that may or may not affect the health of the baby.*

1. If you were offered the choice, which test would you choose?

    ☐ I would choose Test 1

    ☐ I would choose Test 2

    ☐ I would not have an invasive test during pregnancy

    ☐ Don't know

2. Who would you want to make the decision about which test to have?

    ☐ I would want me/me and my partner to make the decision

    ☐ I would want my doctor to make the decision

    ☐ I would want it to be a joint decision with my doctor

    ☐ Don't know

SECTION D: SOME QUESTIONS ABOUT HOW YOU FEEL ABOUT UNCERTAINTY

Please circle the number that best corresponds to how much you agree with each of the following statements:

	Not at all characteristic of me	A little characteristic of me	Somewhat characteristic of me	Very characteristic of me	Entirely characteristic of me
1. Unforeseen events upset me greatly.	1	2	3	4	5
2. It frustrates me not having all the information I need.	1	2	3	4	5
3. Uncertainty keeps me from living a full life.	1	2	3	4	5
4. One should always look ahead so as to avoid surprises.	1	2	3	4	5
5. A small unforeseen event can spoil everything, even with the best of planning.	1	2	3	4	5
6. When it's time to act, uncertainty paralyses me.	1	2	3	4	5
7. When I am uncertain I can't function very well.	1	2	3	4	5
8. I always want to know what the future has in store for me.	1	2	3	4	5
9. I can't stand being taken by surprise.	1	2	3	4	5
10. The smallest doubt can stop me from acting.	1	2	3	4	5
11. I should be able to organize everything in advance.	1	2	3	4	5
12. I must get away from all uncertain situations.	1	2	3	4	5

Questions about your pregnancy

9. Have you had Down syndrome screening in any of your pregnancies?

☐ Yes ☐ No ☐ Don't know

10. Have you had invasive testing in any of your pregnancies?

☐ Yes ☐ No ☐ Don't know

11. Have you had any test results in pregnancy that caused uncertainty about the health of the baby?

☐ Yes ☐ No ☐ Don't know

12. Have you had any test results in pregnancy that indicated that the baby had a genetic condition?

 Yes No Don’t know

13. Have you terminated a pregnancy because something was found during testing (ultrasound or invasive testing) that indicated that the baby had a health issue?

 Yes No Don’t know

14. Are you or any family member affected by a genetic condition?

 Yes No Don’t know

If you would like to discuss any of the issues that have come up, please contact

Antenatal Results and Choices (ARC)

Website: **{** HYPERLINK "http://www.arc-uk.org" **}**

National Helpline: 0845 077 2290 or 0207 713 7486 from a mobile. Monday to Friday, 10.00am-5.30pm

Address: 210 Wandsworth Road, London, SW8 2JU

Email: **{** HYPERLINK "mailto:info@arc-uk.org" **}**
