# Supplementary material for: Factors that impact on women's decision‐making around prenatal genomic tests: An international discrete choice survey
Source: Prenat Diagn. 2022 Apr 30;42(7):934–46. doi: 10.1002/pd.6159 (PMC9325352; doi:10.1002/pd.6159)
Supplement: Supplementary file 2 — Supplementary Material 2 [file PD-42-934-s003.docx]

**Supplementary Figures 2-9**

Supplementary Figure 2: Australia

**Attributes (horizontal text) and levels (vertical text)**

Supplementary Figure 3: China

**Attributes (horizontal text) and levels (vertical text)**

## Supplementary Figure 4: Denmark

**Attributes (horizontal text) and levels (vertical text)**

## Supplementary Figure 5: Netherlands

**Attributes (horizontal text) and levels (vertical text)**

## Supplementary Figure 6: Singapore

**Attributes (horizontal text) and levels (vertical text)**

Supplementary Figure 7: Sweden

**Attributes (horizontal text) and levels (vertical text)**

## Supplementary Figure 8: UK

**Attributes (horizontal text) and levels (vertical text)**

Supplementary Figure 9: USA

**Attributes (horizontal text) and levels (vertical text)**
