# Supplementary material for: Factors that impact on women's decision‐making around prenatal genomic tests: An international discrete choice survey
Source: Prenat Diagn. 2022 Apr 30;42(7):934–46. doi: 10.1002/pd.6159 (PMC9325352; doi:10.1002/pd.6159)
Supplement: Supplementary file 3 — Table S1 [file PD-42-934-s004.docx]

**Supplementary Table 1: Additional summary statistics**

|  | Australia | China | Denmark | Netherlands | Singapore | Sweden | UK | USA | Overall sample |
| --- | --- | --- | --- | --- | --- | --- | --- | --- | --- |
|  | N=178 | N=179 | N=88 | N=177 | N=90 | N=178 | N=174 | N=175 | N=1239 |
| Age in years (mean) | 31.9 | 29.7 | 30.7 | 31.3 | 32.5 | 32.7 | 31.9 | 30.6 | 31.4 |
| Highest educational qualification | | | | | | | | | |
| No or elementary education | 10 (5.6%) | 2 (1.1%) | 5 (5.7%) | 5 (2.8%) | 1 (1.1%) | 6 (3.4%) | 3 (1.7%) | 18 (10.3%) | 50 (4.0%) |
| Lower secondary school education | 16 (9.0%) | 3 (1.7%) | 10 (11.4%) | 21 (11.9%) | 7 (7.8%) | 4 (2.2%) | 18 (10.3%) | 68 (38.9%) | 147 (11.9%) |
| Upper secondary school education | 46 (25.8%) | 19 (10.6%) | 53 (60.2%) | 53 (29.9%) | 18 (20.0%) | 82 (46.1%) | 54 (31.0%) | 32 (18.3%) | 357 (28.8%) |
| Higher education | 106 (59.6%) | 155 (86.6%) | 20 (22.7%) | 98 (55.4%) | 64 (71.1%) | 86 (48.3%) | 99 (56.9%) | 57 (32.6%) | 685 (55.3%) |
| Religious faith | | | | | | | | | |
| None | 78 (44.1%) | 128 (71.5%) | 30 (34.1%) | 85 (48.0%) | 21 (23.3%) | 87 (49.2%) | 76 (43.7%) | 41 (23.4%) | 546 (44.1%) |
| Christian | 62 (35.0%) | 11 (6.1%) | 40 (45.5%) | 67 (37.9%) | 25 (27.8%) | 56 (31.6%) | 76 (43.7%) | 98 (56.0%) | 435 (35.2%) |
| Jewish | 1 (0.6%) | 0 (0.0%) | 0 (0.0%) | 1 (0.6%) | 8 (8.9%) | 1 (0.6%) | 4 (2.3%) | 9 (5.1%) | 24 (1.9%) |
| Muslim | 16 (9.0%) | 0 (0.0%) | 14 (15.9%) | 15 (8.5%) | 11 (12.2%) | 0 (0.0%) | 14 (8.0%) | 8 (4.6%) | 78 (6.3%) |
| Hindu | 12 (6.8%) | 2 (1.1%) | 0 (0.0%) | 5 (2.8%) | 1 (1.1%) | 0 (0.0%) | 2 (1.1%) | 3 (1.7%) | 25 (2.0%) |
| Buddhist | 4 (2.3%) | 35 (19.6%) | 0 (0.0%) | 0 (0.0%) | 24 (26.7%) | 22 (12.4%) | 0 (0.0%) | 2 (1.1%) | 87 (7.0%) |
| Other | 4 (2.3%) | 3 (1.7%) | 4 (4.5%) | 4 (2.3%) | 0 (0.0%) | 5 (2.8%) | 2 (1.1%) | 14 (8.0%) | 36 (2.9%) |
| Rather not answer | 0 (0.0%) | 0 (0.0%) | 0 (0.0%) | 0 (0.0%) | 0 (0.0%) | 6 (3.4%) | 0 (0.0%) | 0 (0.0%) | 6 (0.5%) |
| Ever had Down syndrome screening in a pregnancy | | | | | | | | | |
| Yes | 100 (56.2%) | 125 (69.8%) | 75 (85.2%) | 88 (49.7%) | 52 (57.8%) | 82 (46.1%) | 113 (64.9%) | 86 (49.1%) | 721 (58.2%) |
| No | 71 (39.9%) | 53 (29.6%) | 12 (13.6%) | 81 (45.8%) | 35 (38.9%) | 82 (46.1%) | 53 (30.5%) | 77 (44.0%) | 464 (37.4%) |
| Don't know | 7 (3.9%) | 1 (0.6%) | 1 (1.1%) | 8 (4.5%) | 3 (3.3%) | 14 (7.9%) | 8 (4.6%) | 12 (6.9%) | 54 (4.4%) |
| Ever had invasive testing in any pregnancy | | | | | | | | | |
| Yes | 31 (17.4%) | 100 (55.9%) | 16 (18.2%) | 27 (15.3%) | 23 (25.6%) | 29 (16.3%) | 25 (14.4%) | 41 (23.4%) | 292 (23.6%) |
| No | 134 (75.3%) | 70 (39.1%) | 69 (78.4%) | 141 (79.7%) | 65 (72.2%) | 141 (79.2%) | 135 (77.6%) | 117 (66.9%) | 872 (70.4%) |
| Don't know | 13 (7.3%) | 9 (5.0%) | 3 (3.4%) | 9 (5.1%) | 2 (2.2%) | 8 (4.5%) | 14 (8.0%) | 17 (9.7%) | 75 (6.1%) |
| Ever had test results in pregnancy that showed that the baby had a genetic condition | | | | | | | | | |
| Yes | 27 (15.2%) | 50 (27.9%) | 11 (12.5%) | 12 (6.8%) | 15 (16.7%) | 18 (10.1%) | 22 (12.6%) | 30 (17.1%) | 185 (14.9%) |
| No | 147 (82.6%) | 123 (68.7%) | 73 (83.0%) | 161 (91.0%) | 72 (80.0%) | 153 (86.0%) | 141 (81.0%) | 135 (77.1%) | 1005 (81.1%) |
| Don't know | 4 (2.2%) | 6 (3.4%) | 4 (4.5%) | 4 (2.3%) | 3 (3.3%) | 7 (3.9%) | 11 (6.3%) | 10 (5.7%) | 49 (4.0%) |
| Ever terminated a pregnancy because the baby had a health issue | | | | | | | | | |
| Yes | 14 (7.9%) | 40 (22.3%) | 7 (8.0%) | 11 (6.2%) | 6 (6.7%) | 18 (10.1%) | 17 (9.8%) | 22 (12.6%) | 135 (10.9%) |
| No | 159 (89.3%) | 134 (74.9%) | 77 (87.5%) | 163 (92.1%) | 81 (90.0%) | 154 (86.5%) | 150 (86.2%) | 149 (85.1%) | 1067 (86.1%) |
| Don't know | 5 (2.8%) | 5 (2.8%) | 4 (4.5%) | 3 (1.7%) | 3 (3.3%) | 6 (3.4%) | 7 (4.0%) | 4 (2.3%) | 37 (3.0%) |
| Total children (mean) | 1.9 | 1.3 | 1.7 | 1.8 | 1.7 | 1.9 | 1.9 | 2.0 | 1.8 |
| Months since last baby was born (mean) | 11.5 | 9.1 | 11.8 | 11.4 | 11.1 | 11.2 | 14.5 | 9.2 | 11.2 |
| Hypothetical question: if you were offered the choice, which test would you choose? | | | | | | | | | |
| Test 1 (targeted) | 44 (24.7%) | 91 (50.8%) | 21 (23.9%) | 41 (23.2%) | 27 (30.0%) | 33 (18.5%) | 51 (29.3%) | 41 (23.4%) | 349 (28.2%) |
| Test 2 (broad) | 91 (51.1%) | 76 (42.5%) | 50 (56.8%) | 81 (45.8%) | 49 (54.4%) | 92 (51.7%) | 81 (46.6%) | 87 (49.7%) | 607 (49.0%) |
| Neither | 28 (15.7%) | 10 (5.6%) | 11 (12.5%) | 40 (22.6%) | 10 (11.1%) | 41 (23.0%) | 32 (18.4%) | 37 (21.1%) | 209 (16.9%) |
| Don't know | 15 (8.4%) | 2 (1.1%) | 6 (6.8%) | 15 (8.5%) | 4 (4.4%) | 12 (6.7%) | 10 (5.7%) | 10 (5.7%) | 74 (6.0%) |
| Hypothetical question: who would you want to make the decision about which test to have? | | | | | | | | | |
| I would want me/me and my partner to make the decision | 98 (55.1%) | 115 (64.2%) | 50 (56.8%) | 107 (60.5%) | 58 (64.4%) | 98 (55.1%) | 100 (57.5%) | 95 (54.3%) | 721 (58.2%) |
| I would want my doctor to make the decision | 23 (12.9%) | 20 (11.2%) | 27 (30.7%) | 12 (6.8%) | 10 (11.1%) | 14 (7.9%) | 22 (12.6%) | 23 (13.1%) | 151 (12.2%) |
| I would want it to be a joint decision with my doctor | 46 (25.8%) | 44 (24.6%) | 7 (8.0%) | 52 (29.4%) | 22 (24.4%) | 60 (33.7%) | 43 (24.7%) | 51 (29.1%) | 325 (26.2%) |
| Don't know | 11 (6.2%) | 0 (0.0%) | 4 (4.5%) | 6 (3.4%) | 0 (0.0%) | 6 (3.4%) | 9 (5.2%) | 6 (3.4%) | 1. 3.4%) |
| How religious are you | | | | | | | | | |
| Not very religious | 113 (63.5%) | 42 (23.5%) | 56 (63.6%) | 121 (69.5%) | 46 (51.1%) | 107 (60.5%) | 113 (64.9%) | 78 (44.6%) | 676 (54.7%) |
| Quite religious | 46 (25.8%) | 69 (38.5%) | 16 (18.2%) | 44 (25.3%) | 35 (38.9%) | 44 (24.9%) | 47 (27.0%) | 65 (37.1%) | 366 (29.6%) |
| Very religious | 19 (10.7%) | 68 (38.0%) | 16 (18.2%) | 9 (5.2%) | 9 (10.0%) | 26 (14.7%) | 14 (8.0%) | 32 (18.3%) | 193 (15.6%) |
| Intolerance for Uncertainty Scale (mean)* | 35.9 | 36.6 | 33.4 | 32.6 | 40.2 | 36.2 | 37.1 | 35.3 | 35.8 |

*Note: Each item on the Intolerance for Uncertainty Scale is scored from 1 (not at all characteristic of me) to 5 (entirely characteristic of me) with higher scores indicating lower intolerance for uncertainty. The minimum score is 12 and the maximum score is 60.
