# Supplementary material for: Factors that impact on women's decision‐making around prenatal genomic tests: An international discrete choice survey
Source: Prenat Diagn. 2022 Apr 30;42(7):934–46. doi: 10.1002/pd.6159 (PMC9325352; doi:10.1002/pd.6159)
Supplement: Supplementary file 4 — Table S2 [file PD-42-934-s001.docx]

**Supplementary Table 2: Quantitative ranking results**

|  | Australia | China | Denmark | Netherlands | Singapore | Sweden | UK | USA | Overall sample |
| --- | --- | --- | --- | --- | --- | --- | --- | --- | --- |
|  | N=178 | N=179 | N=88 | N=177 | N=90 | N=178 | N=174 | N=175 | N=1239 |
| Ranking - likelihood of getting a diagnosis | | | | | | | | | |
| 1 (Most important) | 57 (32.0%) | 63 (35.2%) | 36 (40.9%) | 35 (19.8%) | 22 (24.4%) | 45 (25.3%) | 60 (34.5%) | 53 (30.3%) | 371 (29.9%) |
| 2 | 68 (38.2%) | 44 (24.6%) | 30 (34.1%) | 88 (49.7%) | 31 (34.4%) | 74 (41.6%) | 69 (39.7%) | 59 (33.7%) | 463 (37.4%) |
| 3 | 28 (15.7%) | 28 (15.6%) | 11 (12.5%) | 30 (16.9%) | 20 (22.2%) | 33 (18.5%) | 27 (15.5%) | 29 (16.6%) | 206 (16.6%) |
| 4 | 7 (3.9%) | 18 (10.1%) | 5 (5.7%) | 14 (7.9%) | 7 (7.8%) | 16 (9.0%) | 10 (5.7%) | 19 (10.9%) | 96 (7.7%) |
| 5 | 10 (5.6%) | 14 (7.8%) | 5 (5.7%) | 7 (4.0%) | 5 (5.6%) | 4 (2.2%) | 5 (2.9%) | 9 (5.1%) | 59 (4.8%) |
| 6 (Least important) | 8 (4.5%) | 12 (6.7%) | 1 (1.1%) | 3 (1.7%) | 5 (5.6%) | 6 (3.4%) | 3 (1.7%) | 6 (3.4%) | 44 (3.6%) |
| Ranking - type of health professional | | | | | | | | | |
| 1 (Most important) | 13 (7.3%) | 35 (19.6%) | 9 (10.2%) | 12 (6.8%) | 15 (16.7%) | 13 (7.3%) | 17 (9.8%) | 42 (24.0%) | 156 (12.6%) |
| 2 | 38 (21.3%) | 48 (26.8%) | 13 (14.8%) | 20 (11.3%) | 17 (18.9%) | 31 (17.4%) | 32 (18.4%) | 24 (13.7%) | 223 (18.0%) |
| 3 | 38 (21.3%) | 37 (20.7%) | 20 (22.7%) | 38 (21.5%) | 24 (26.7%) | 40 (22.5%) | 37 (21.3%) | 34 (19.4%) | 268 (21.6%) |
| 4 | 30 (16.9%) | 29 (16.2%) | 19 (21.6%) | 30 (16.9%) | 18 (20.0%) | 28 (15.7%) | 28 (16.1%) | 29 (16.6%) | 211 (17.0%) |
| 5 | 32 (18.0%) | 25 (14.0%) | 10 (11.4%) | 32 (18.1%) | 10 (11.1%) | 27 (15.2%) | 26 (14.9%) | 24 (13.7%) | 186 (15.0%) |
| 6 (Least important) | 27 (15.2%) | 5 (2.8%) | 17 (19.3%) | 45 (25.4%) | 6 (6.7%) | 39 (21.9%) | 34 (19.5%) | 22 (12.6%) | 195 (15.7%) |
| Ranking - test safety | | | | | | | | | |
| 1 (Most important) | 93 (52.2%) | 56 (31.3%) | 34 (38.6%) | 111 (62.7%) | 39 (43.3%) | 97 (54.5%) | 86 (49.4%) | 63 (36.0%) | 579 (46.7%) |
| 2 | 31 (17.4%) | 34 (19.0%) | 23 (26.1%) | 23 (13.0%) | 14 (15.6%) | 31 (17.4%) | 38 (21.8%) | 40 (22.9%) | 234 (18.9%) |
| 3 | 25 (14.0%) | 51 (28.5%) | 15 (17.0%) | 23 (13.0%) | 17 (18.9%) | 26 (14.6%) | 32 (18.4%) | 32 (18.3%) | 221 (17.8%) |
| 4 | 20 (11.2%) | 19 (10.6%) | 11 (12.5%) | 7 (4.0%) | 10 (11.1%) | 12 (6.7%) | 8 (4.6%) | 30 (17.1%) | 117 (9.4%) |
| 5 | 4 (2.2%) | 15 (8.4%) | 2 (2.3%) | 9 (5.1%) | 5 (5.6%) | 7 (3.9%) | 5 (2.9%) | 5 (2.9%) | 52 (4.2%) |
| 6 (Least important) | 5 (2.8%) | 4 (2.2%) | 3 (3.4%) | 4 (2.3%) | 5 (5.6%) | 5 (2.8%) | 5 (2.9%) | 5 (2.9%) | 36 (2.9%) |
| Ranking - waiting time for results | | | | | | | | | |
| 1 (Most important) | 6 (3.4%) | 7 (3.9%) | 2 (2.3%) | 7 (4.0%) | 6 (6.7%) | 8 (4.5%) | 4 (2.3%) | 9 (5.1%) | 49 (4.0%) |
| 2 | 19 (10.7%) | 13 (7.3%) | 8 (9.1%) | 23 (13.0%) | 11 (12.2%) | 11 (6.2%) | 11 (6.3%) | 21 (12.0%) | 117 (9.4%) |
| 3 | 34 (19.1%) | 24 (13.4%) | 15 (17.0%) | 41 (23.2%) | 11 (12.2%) | 21 (11.8%) | 33 (19.0%) | 40 (22.9%) | 219 (17.7%) |
| 4 | 42 (23.6%) | 67 (37.4%) | 20 (22.7%) | 49 (27.7%) | 16 (17.8%) | 52 (29.2%) | 54 (31.0%) | 37 (21.1%) | 337 (27.2%) |
| 5 | 41 (23.0%) | 27 (15.1%) | 22 (25.0%) | 36 (20.3%) | 23 (25.6%) | 55 (30.9%) | 37 (21.3%) | 38 (21.7%) | 279 (22.5%) |
| 6 (Least important) | 36 (20.2%) | 41 (22.9%) | 21 (23.9%) | 21 (11.9%) | 23 (25.6%) | 31 (17.4%) | 35 (20.1%) | 30 (17.1%) | 238 (19.2%) |
| Ranking - secondary findings reported | | | | | | | | | |
| 1 (Most important) | 2 (1.1%) | 2 (1.1%) | 1 (1.1%) | 3 (1.7%) | 0 (0.0%) | 5 (2.8%) | 5 (2.9%) | 4 (2.3%) | 22 (1.8%) |
| 2 | 8 (4.5%) | 11 (6.1%) | 5 (5.7%) | 7 (4.0%) | 5 (5.6%) | 17 (9.6%) | 7 (4.0%) | 14 (8.0%) | 74 (6.0%) |
| 3 | 31 (17.4%) | 21 (11.7%) | 13 (14.8%) | 16 (9.0%) | 6 (6.7%) | 38 (21.3%) | 28 (16.1%) | 18 (10.3%) | 171 (13.8%) |
| 4 | 39 (21.9%) | 26 (14.5%) | 11 (12.5%) | 42 (23.7%) | 23 (25.6%) | 43 (24.2%) | 38 (21.8%) | 34 (19.4%) | 256 (20.7%) |
| 5 | 61 (34.3%) | 77 (43.0%) | 38 (43.2%) | 66 (37.3%) | 30 (33.3%) | 53 (29.8%) | 67 (38.5%) | 60 (34.3%) | 452 (36.5%) |
| 6 (Least important) | 37 (20.8%) | 42 (23.5%) | 20 (22.7%) | 43 (24.3%) | 26 (28.9%) | 22 (12.4%) | 29 (16.7%) | 45 (25.7%) | 264 (21.3%) |
| Ranking - uncertain results reported | | | | | | | | | |
| 1 (Most important) | 7 (3.9%) | 16 (8.9%) | 6 (6.8%) | 9 (5.1%) | 8 (8.9%) | 10 (5.6%) | 2 (1.1%) | 4 (2.3%) | 62 (5.0%) |
| 2 | 14 (7.9%) | 29 (16.2%) | 9 (10.2%) | 16 (9.0%) | 12 (13.3%) | 14 (7.9%) | 17 (9.8%) | 17 (9.7%) | 128 (10.3%) |
| 3 | 22 (12.4%) | 18 (10.1%) | 14 (15.9%) | 29 (16.4%) | 12 (13.3%) | 20 (11.2%) | 17 (9.8%) | 22 (12.6%) | 154 (12.4%) |
| 4 | 40 (22.5%) | 20 (11.2%) | 22 (25.0%) | 35 (19.8%) | 16 (17.8%) | 27 (15.2%) | 36 (20.7%) | 26 (14.9%) | 222 (17.9%) |
| 5 | 30 (16.9%) | 21 (11.7%) | 11 (12.5%) | 27 (15.3%) | 17 (18.9%) | 32 (18.0%) | 34 (19.5%) | 39 (22.3%) | 211 (17.0%) |
| 6 (Least important) | 65 (36.5%) | 75 (41.9%) | 26 (29.5%) | 61 (34.5%) | 25 (27.8%) | 75 (42.1%) | 68 (39.1%) | 67 (38.3%) | 462 (37.3%) |
| *Average ranking (mean)* | | | | | | | | | |
| Ranking - likelihood of getting a diagnosis | *2.3 (1.3)* | *2.5 (1.6)* | *2.0 (1.2)* | *2.3 (1.1)* | *2.5 (1.4)* | *2.3 (1.2)* | *2.1 (1.1)* | *2.4 (1.3)* | *2.3 (1.3)* |
| Ranking - type of health professional | *3.6 (1.5)* | *2.9 (1.4)* | *3.7 (1.6)* | *4.0 (1.6)* | *3.1 (1.5)* | *3.8 (1.6)* | *3.7 (1.6)* | *3.2 (1.7)* | *3.5 (1.6)* |
| Ranking - test safety | *2.0 (1.3)* | *2.5 (1.4)* | *2.2 (1.3)* | *1.8 (1.3)* | *2.4 (1.5)* | *2.0 (1.3)* | *2.0 (1.3)* | *2.4 (1.3)* | *2.1 (1.4)* |
| Ranking - waiting time for results | *4.1 (1.4)* | *4.2 (1.3)* | *4.3 (1.4)* | *3.8 (1.3)* | *4.2 (1.6)* | *4.3 (1.3)* | *4.2 (1.3)* | *3.9 (1.4)* | *4.1 (1.4)* |
| Ranking - secondary findings reported | *4.5 (1.2)* | *4.6 (1.2)* | *4.6 (1.2)* | *4.6 (1.2)* | *4.7 (1.1)* | *4.1 (1.3)* | *4.4 (1.2)* | *4.5 (1.3)* | *4.5 (1.2)* |
| Ranking - uncertain results reported | *4.5 (1.5)* | *4.3 (1.8)* | *4.1 (1.6)* | *4.3 (1.6)* | *4.1 (1.7)* | *4.6 (1.6)* | *4.6 (1.4)* | *4.6 (1.5)* | - 1. *(1.6)* |
