# Supplementary material for: Factors that impact on women's decision‐making around prenatal genomic tests: An international discrete choice survey
Source: Prenat Diagn. 2022 Apr 30;42(7):934–46. doi: 10.1002/pd.6159 (PMC9325352; doi:10.1002/pd.6159)
Supplement: Supplementary file 5 — Table S3 [file PD-42-934-s002.docx]

**Supplementary Table 3: Standard deviations**

|  | AUSTRALIA | CHINA | DENMARK | NETHERLANDS | SINGAPORE | SWEDEN | UK | USA | All |
| --- | --- | --- | --- | --- | --- | --- | --- | --- | --- |
|  | **N=178** | **N=179** | **N=88** | **N=177** | **N=90** | **N=178** | **N=174** | **N=175** | **N=1239** |
| **5 out of every 100 cases (5% of cases)** | 1.101*** (0.101) | 0.467*** (0.056) | 1.599*** (0.199) | 1.178*** (0.109) | 1.677*** (0.217) | 1.387*** (0.124) | 1.110*** (0.107) | 0.830*** (0.081) | -1.081*** (0.038) |
| **30 out of every 100 cases (30% of cases)** | -0.081 (0.263) | 0.009 (0.080) | 0.002 (0.116) | -0.066 (0.139) | 0.497*** (0.112) | -0.002 (0.108) | 0.006 (0.281) | 0.001 (0.161) | 0.001 (0.054) |
| **1 week** | 0.335*** (0.070) | -0.002 (0.108) | 0.450*** (0.108) | 0.338*** (0.074) | -0.394*** (0.108) | -0.291*** (0.087) | -0.172 (0.095) | 0.162 (0.088) | 0.216*** (0.032) |
| **2 weeks** | -0.004 (0.108) | 0.005 (0.057) | -0.110 (0.185) | 0.032 (0.132) | -0.035 (0.125) | 0.095 (0.142) | -0.026 (0.224) | 0.000 (0.076) | 0.019 (0.039) |
| **Genetic specialist with specialist knowledge of the test findings but who you have not met before** | -0.160** (0.056) | 0.181*** (0.042) | 0.266** (0.083) | 0.246*** (0.052) | -0.310*** (0.082) | 0.207*** (0.055) | 0.132* (0.062) | 0.166** (0.053) | -0.187*** (0.020) |
| **Uncertain results reported back to parents** | 0.275*** (0.050) | 0.145** (0.047) | -0.208* (0.100) | 0.396*** (0.055) | 0.492*** (0.095) | 0.284*** (0.054) | 0.307*** (0.052) | 0.269*** (0.048) | 0.272*** (0.019) |
| **Secondary findings reported back to parents** | -0.226*** (0.054) | 0.031 (0.128) | 0.483*** (0.093) | 0.314*** (0.050) | 0.282*** (0.080) | 0.320*** (0.053) | 0.101 (0.108) | 0.167** (0.054) | -0.217*** (0.020) |
| **Alternative specific constant (Opt-out)** | 3.286*** (0.400) | 1.390** (0.513) | 3.532*** (0.480) | 3.181*** (0.327) | 3.764*** (0.584) | 2.746*** (0.305) | -2.619*** (0.301) | 2.160*** (0.240) | 2.904*** (0.136) |

**=p<0.1; **=p<0.05; ***=p<0.01.* Note: Standard errors in parentheses.
